# Patient-centered outcomes after surgical treatment of peri-implantitis: a prospective clinical study

**DOI:** 10.4317/medoral.25587

**Published:** 2022-10-16

**Authors:** Octavi Camps-Font, Irene Pérez-Beltrán, Vicente Fornés-Nieto, Albert González-Barnadas, Xavier Costa-Berenguer, Marta García-García, Rui Figueiredo, Eduard Valmaseda-Castellón

**Affiliations:** 1 DDS, MS, PhD. Assistant Professor of Oral Surgery and Professor of the Master of Oral Surgery and Implantology degree program. Faculty of Medicine and Health Sciences, University of Barcelona, Barcelona, Spain. Researcher of the IDIBELL Research Institute, Barcelona, Spain; 2 DDS, MS. Master of Oral Surgery and Implantology degree program. Faculty of Medicine and Health Sciences, University of Barcelona, Barcelona, Spain; 3 DDS, MS. Professor of the Master of Oral Surgery and Implantology degree program. Faculty of Medicine and Health Sciences, University of Barcelona, Barcelona, Spain; 4 DDS, MS, PhD. Assistant Professor of the Dental degree and Professor of the Master of Oral Surgery and Implantology degree program. Faculty of Medicine and Health Sciences, University of Barcelona, Barcelona (Spain). Researcher of the IDIBELL Research Institute, Barcelona, Spain; 5 DDS, MS, PhD. Associate Professor of Oral Surgery and Professor of the Master of Oral Surgery and Implantology degree program. Faculty of Medicine and Health Sciences, University of Barcelona. Researcher at the IDIBELL Institute, Barcelona, Spain; 6 DDS, MS, PhD. Full Professor of Oral Surgery. Director of the Master of Oral Surgery and Implantology degree program. Faculty of Medicine and Health Sciences, University of Barcelona. Researcher at the IDIBELL Institute, Barcelona, Spain

## Abstract

**Background:**

Peri-implantitis is an inflammatory process affecting soft and hard tissues surrounding dental implants, causing progressive marginal bone loss. Peri-implant surgery is the treatment of choice. However, evidence about its impact on patients’ quality of life (QoL) is limited. This study aimed to assess pain and QoL in the first seven post-operative days and measure patient satisfaction at the end of this period.

**Material and Methods:**

A prospective cohort study was conducted in patients with peri-implantitis. Patients reported pain on a visual analogue scale (VAS) ranging from 0 to 100mm every day during the first week after surgery. They then completed the OHIP-14sp questionnaire. A descriptive and inferential data analysis was used to assess the effect of surgical approach (resective, regenerative or combined), gender and working status on pain, satisfaction and QoL.

**Results:**

Forty-one patients (93,2%) completed the daily pain VAS; scores ranged from 0 to 95 mm. Gender, occupation, or type of surgery had no significant effect upon its evolution. The mean total OHIP-14sp score was 16.7 (range = 5 to 33), indicating low to moderate deterioration in perceived oral health. Postoperative OHRQoL was significantly higher in working patients (mean difference (MD): 3.94; *P* = 0.042), and with the regenerative (MD: 6.34; *P* = 0.044) or the combined approach (MD: 5.41; *P* = 0.027).

**Conclusions:**

Considering the limitations of this study, postoperative pain was mild to moderate and decreased after the third day. Surgical treatment of peri-implantitis has an impact on QoL, especially when augmentation procedures are involved. This impact is higher in working patients.

** Key words:**Patient-centered outcomes, quality of life, peri-implantitis, peri-implant surgery, dental implants.

## Introduction

Over recent decades, the use of dental implants for oral rehabilitation has been proven to be a reliable therapy due to its apparent predictability (1). However, as the global number of patients that receive implant-supported restorations is increasing, biological complications (i.e. mucositis and peri-implantitis) have become a common problem in daily clinical practice. Reports in the literature indicate that the prevalence of peri-implantitis ranges from 9.3% to 12.8% of implants and from 18.5% to 19.8% of patients (2,3). Since non-surgical peri-implantitis therapy seems to be ineffective, surgery has become the treatment of choice (4). These surgical procedures commonly involve open flap debridement, removal of the granulation tissue and cleaning of the exposed surface of the implant. Subsequently, bone grafting, implantoplasty and bone resection or a combination of both might be performed (5,6).

Clinical outcomes mainly evaluate inflammation and progression of bone loss and do not consider the patient’s opinion. Knowing the impact of these procedures from the patient’s perspective is of great importance in assessing the overall success of the treatment (7). The concept of oral health-related quality of life (OHRQoL) has gained in popularity and attracted greater attention in recent years (8). However, despite this growing tendency to report patient-centered outcomes, to the present authors’ knowledge the available evidence regarding the impact of peri-implant surgery on OHRQoL is still scarce (9). Thus, the aim of the present study was to describe the self-reported postoperative pain and assess the OHRQoL and degree of satisfaction during the first postoperative week of patients undergoing surgical treatment for peri-implantitis.

## Material and Methods

A prospective cohort study was conducted in consecutive patients receiving surgical treatment for peri-implantitis in the course of the Master’s Degree Program in Oral Surgery and Implantology at the University of Barcelona (Barcelona, Spain). The protocol was approved by the Ethics Committee (CEIm) of the University of Barcelona Dental Hospital (2014/20) and the study was conducted in accordance with the Declaration of Helsinki on human studies. The STROBE Statement guidelines for cross-sectional studies were applied in the study design and data reporting (10). Individuals were given full information about the surgical procedures and signed an informed consent form.

- Case definition of peri-implantitis

Preoperative analysis included complete medical histories and clinical examination, and examination of periapical radiographs taken with a positioner using the long-cone paralleling technique. Patients with peri-implantitis according to the definition of the 2017 World Workshop on the Classification of Periodontal and Peri-Implant Diseases and Conditions (11) were included, as follows:

- Patients with implants with bleeding on probing and/or suppuration; increased probing pocket depths and progressive bone loss.

- In cases without previous records, implants with bleeding or suppuration on gentle probing, PPD 6mm and bone level 3mm apical to the most coronal part of the rough surface of the implant.

If a patient required multiple surgeries, only the first one was recorded.

The exclusion criteria were general contraindications to implant surgery, radiotherapy in the head and neck area, uncontrolled diabetes, current pregnancy or lactation, alcohol abuse and previous history of peri-implant surgical treatment. Patients were excluded if they did not complete the postoperative pain visual analog scales (VAS) or the OHRQoL questionnaire.

- Management of peri-implantitis

**Figure 1:**
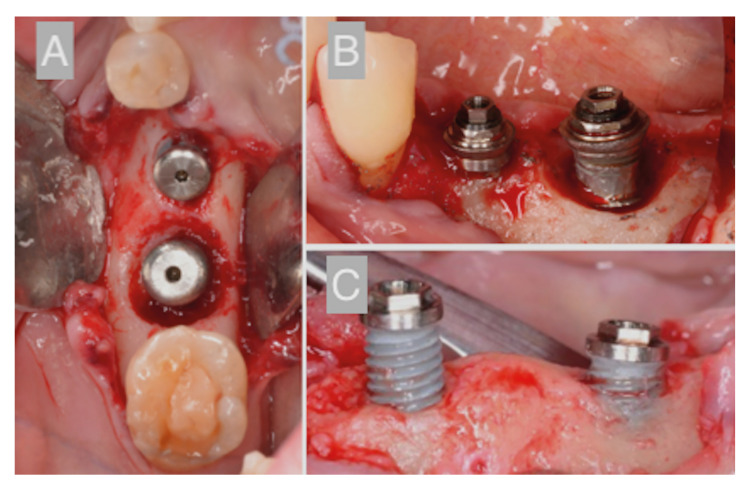
Peri-implant defect anatomy and its relation with the different surgical approaches. A) Class E defect that usually requires a regenerative approach; B) Class E and Class II defect which needs a combined approach; C) Class II defect, which frequently needs a resective approach.

All treatments were performed by fellows in the Master’s Degree Program in Oral Surgery and Implantology at the University of Barcelona under the direct supervision of a consultant surgeon and a periodontist. All the patients underwent nonsurgical peri-implant therapy 6 weeks prior to surgery, by means of scaling and debridement of the peri-implant sulcus. In implants with no access to oral hygiene, implant-supported prosthesis was modified to favor the use of interproximal brush (12). The surgical procedure applied depended on the peri-implant bone defect morphology, as described by Schwarz *et al*. (13): a) a resective approach by means of an apically repositioned flap with supracrestal implantoplasty of the exposed implants was used for Class II and Ia defects; b) a regenerative technique using natural bone mineral of bovine origin (BioOss® spongiosa granules, particle size 0.25-1 mm; Geistlich, Wolhusen, Switzerland) covered with a collagen membrane (BioGide®; Geistlich, Wolhusen, Switzerland) was employed for Class Ie; and c) a combined approach with buccal or supracrestal implantoplasty and regenerative techniques was used for combined defects (Class II + Ib/Ic/Ie) (Fig. 1).

Written postoperative instructions and drug prescriptions were given to the patients after the surgical procedure. All patients were prescribed 750 mg of amoxicillin every 8 hours for 7 days and 600 mg of ibuprofen every 8 hours for 5 days. If needed, patients were instructed to take 1g paracetamol. They were also instructed to rinse twice daily for 2 minutes with 0.12% chlorhexidine digluconate with 0.05% cetylpyridinium chloride, for two weeks.

- Data sampling

A trained researcher (I.P-B.) collected all the data. This included age, gender, educational level (primary school, high school or university), occupation, location (maxilla or mandible), position (anterior, posterior or both), number of implants treated, peri-implant probing depth without prosthesis assessed at 6 sites per implant on gentle probing (all prostheses were removed) and surgical approach (resective, regenerative or combined).

After surgery, the patients were asked to fill in a pain questionnaire every 24h during the first week. Pain was measured on a 100 mm visual analogue scale (VAS), with 0 mm representing “no pain” and 100 mm “worst pain imaginable”. The participants were also asked to report the daily number of rescue Tablets they took.

Seven days after the peri-implant surgical treatment, all the patients completed the Spanish version of the Oral Health Impact Profile questionnaire (OHIP-14sp) (14). OHIP-14sp consists of 14 questions covering seven domains (functional limitation, physical pain, psychological discomfort, physical disability, psychological disability, social disability, and handicap). The participants answered every item on 5-point Likert scales ranging from 0 to 4 (never or not applicable = 0, hardly ever = 1, occasionally = 2, fairly often = 3 and very often = 4). "Don't know" and blank entries were entered as missing values. Accordingly, the possible total scores ranged from 0 to 56; higher scores indicate a poorer OHRQoL (15). Seven days after the peri-implant surgical treatment, they also completed a Postsurgical Patient Satisfaction Questionnaire (PSPSQ) to assess the short-term patient satisfaction (16).

- Statistical analysis

The statistical analysis was carried out with Stata14 (StataCorp®, College Station, TX, USA).

The postoperative VAS score was the primary outcome variable, while OHIP-14sp and PSPSQ scores were considered the secondary outcome variables. Categorical outcomes were presented as absolute and relative frequencies. The groups were compared through bivariate analysis with Chi-square tests, or Fisher’s exact tests when Chi-square test assumptions were not fulfilled. Normality of scale variables was explored using the Shapiro-Wilk test and visual analysis of the P-P plot and box plot.

Where normality was rejected, the interquartile range (IQR) and median were calculated. Where the distribution was compatible with normality, the mean and standard deviation (SD) were used. Differences between groups of scale variables were explored using parametric tests (Student’s t test for independent samples or one-way analysis of variance (ANOVA)), or nonparametric tests (Mann-Whitney U-test or Kruskal-Wallis test).

To analyze the influence of gender, educational level, working status, surgical approach, and number of implants on pain evolution, a repeated measures mixed model for each categorical covariate was built. Fulfillment of the assumptions was checked by means of the graphical distribution of the residuals.

The reliability of each questionnaire was assessed with the Cronbach α coefficient. The level of significance was set at *P* < 0.05, using Bonferroni’s correction for multiplicity of contrasts. To determine the power for surgical approach and OHRQoL, post-hoc ANOVA using Cohen’s ƒ2 test was performed.

**Table 1:**
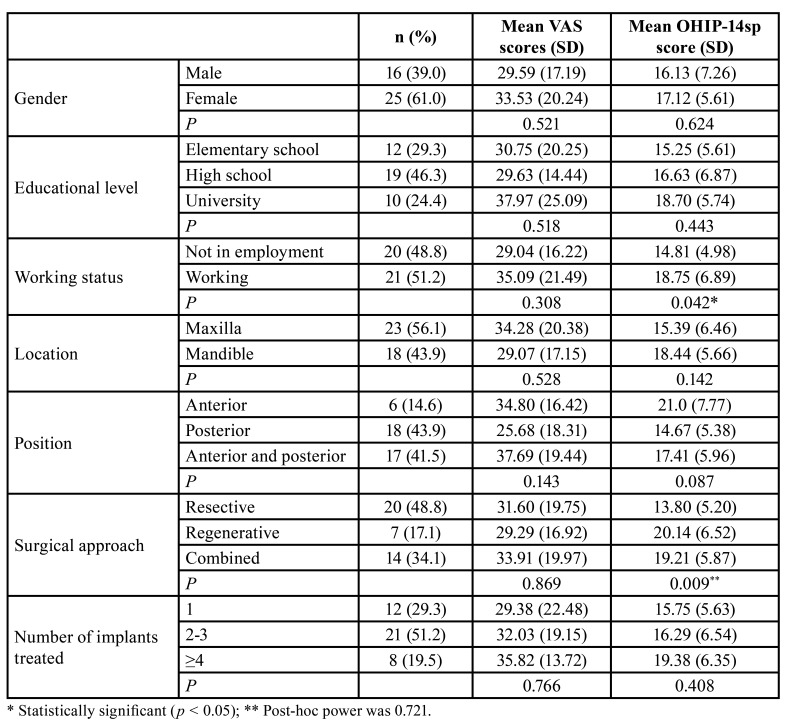
Sample distribution, Pain intensity on a visual analogue scale (VAS) and Oral Health Impact Profile questionnaire (OHIP-14sp) scores.

## Results

Of 45 patients treated with peri-implantitis surgery, 41 (81.1%) completed the questionnaire. The mean age of the participants was 61.5 years ± 10.5. The majority of the patients included were women (61.0%). Mean peri-implant probing depth was 4.9 mm ± 2.0. Table 1 shows demographic data.

**Figure 2:**
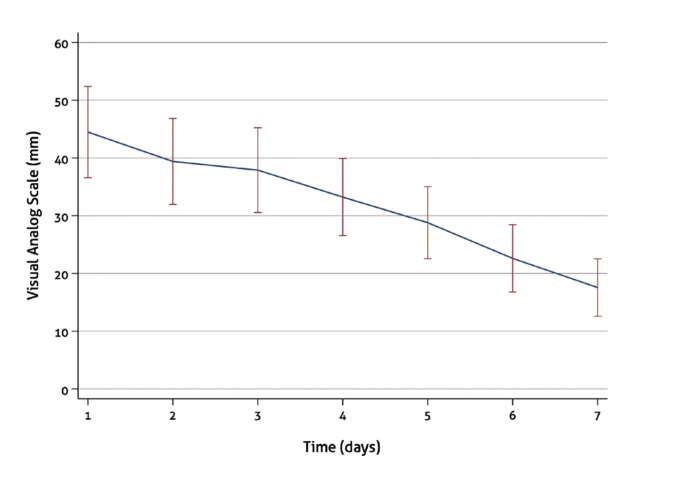
Visual analog scale (VAS) of mean pain scores on the 7 days after peri-implant surgical treatment.

VAS pain scores decreased significantly over time (F = 36.08; df = 6; *P* < 0.001). Daily VAS scores with a 95% confidence interval (CI) are shown in Fig. 2. No significant differences were observed during the first 3 days. However, from day 4 to 7 the pain scores decreased significantly (day 1 vs. days 4 to 7: *P* < 0.001; day 2 vs. days 5 to 7: *P* < 0.001; day 3 vs. 5 to 7: *P* ≤ 0.002; day 4 vs. day 6 and 7: *P* < 0.001; and day 5 vs. day 7: *P* < 0.001). The VAS pain scores did not differ between any of the grouping levels (*P* ≥ 0.05) (Table 1) and the decrease rate was similar in all the groups (*P* ≥ 0.05).

The percentage of patients who took analgesics on each day and the mean number of days of analgesic intake are shown in Table 2.

**Table 2:**
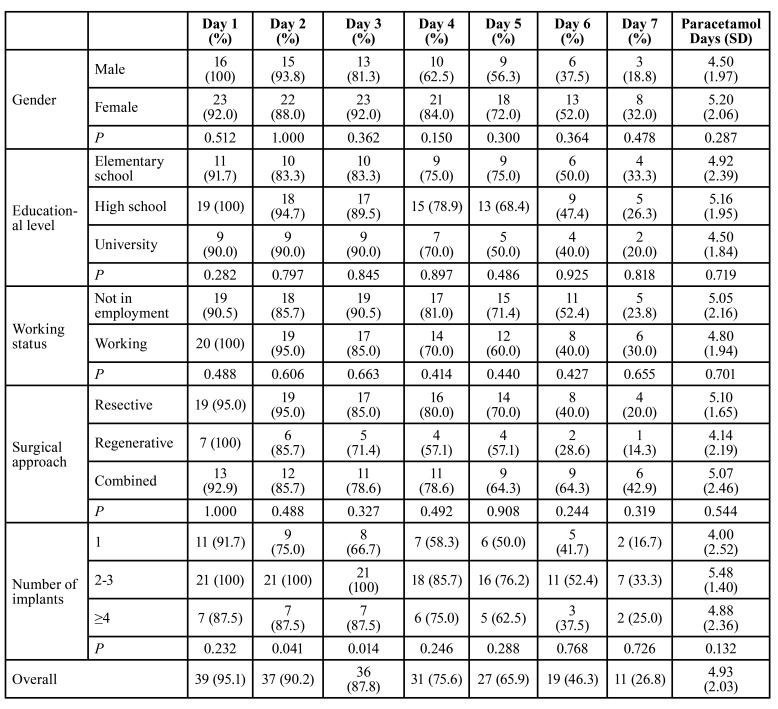
Percentage of participants who took rescue medication (paracetamol).

All the groups soon discontinued the use of paracetamol. Patients who had a single implant treated took significantly more analgesics on days 2 and 3 than patients who required treatment to several implants (*P* = 0.041 for 2-3 implants and *P* = 0.014 for ≥4 implants).

The impact of peri-implant surgical therapy on OHIP-14sp is reported in Table 3. The mean total OHIP-14sp score was 16.7 ± 6.2, indicating a low to moderate deterioration in perceived oral health. The lowest mean value was for the handicap domain (0.7± 0.8), while the highest was for the physical pain domain (2.0±1.1). Postoperative OHRQoL was mainly influenced by working status, since working participants returned higher scores than those who were not in employment (MD: 3.94 points; 95%CI: 0.16 to 7.72; *P* = 0.042).

**Table 3:**
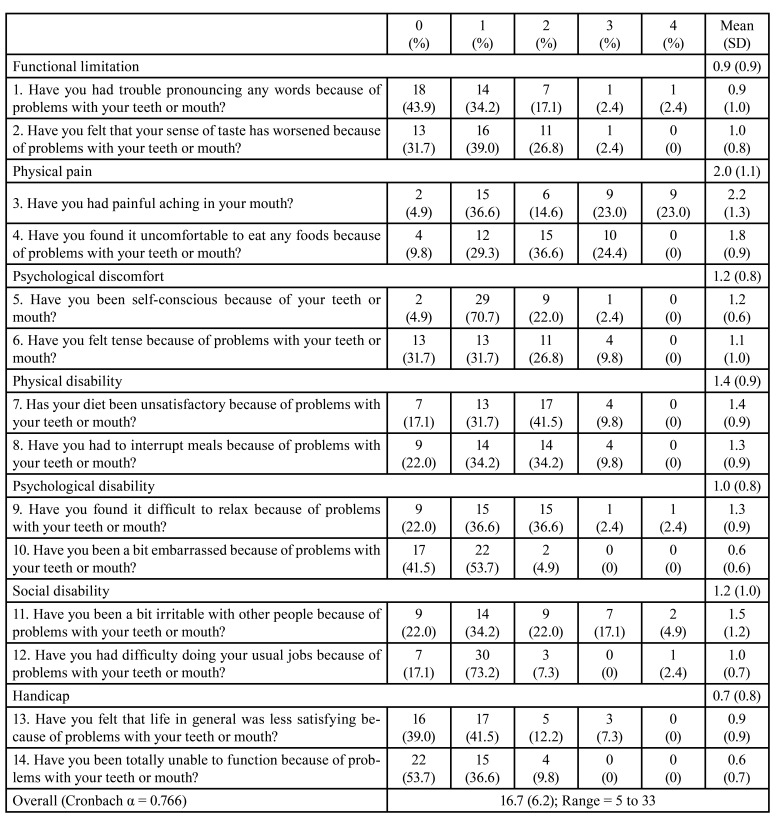
Oral Health Impact Profile questionnaire (OHIP-14) in English, This study used the validated translation into Spanish (OHIP-14sp) (n = 41).

Similarly, patients treated with a regenerative or a combined approach reported higher OHIP-14sp scores than patients undergoing resective surgery (regenerative vs. resective MD: 6.34 points; 95%CI: 0.12 to 12.57; *P* = 0.044; combined vs. resective MD: 5.41 points; 95%CI: 0.48 to 10.35; *P* = 0.027) (Table 1).

Regarding PSPSQ, 78.0% of patients were satisfied with the treatment and 87.8% would recommend it. However, 36.6% would not repeat it and only 51.2% felt that their problem had been solved. Almost a third of the patients reported the maximal level of satisfaction (Table 4) (16).

**Table 4:**
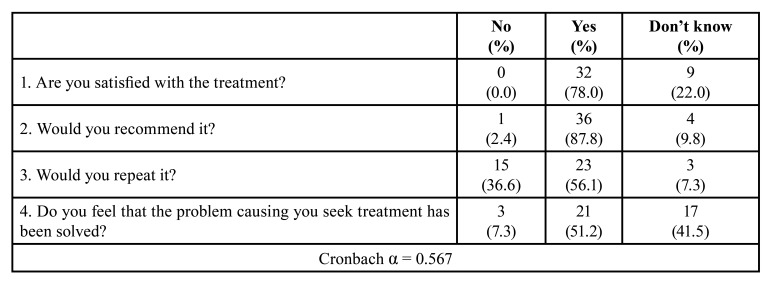
Patient Satisfaction Questionnaire (n = 41).

## Discussion

As with any other oral surgical procedure, the treatment of peri-implantitis had an impact on the patient’s quality of life. The participants reported moderate pain during the first 3 days, which decreased progressively from the fourth day. The mean total OHIP-14sp score was 16.7, and the subjects frequently reported an impact on their quality of life in the physical pain and physical disability domains. Approximately 80% of the patients were satisfied with the treatment but one third of the participants would not repeat it.

To the best of authors’ knowledge, this is the first study to have evaluated changes in the quality of life during the first postoperative week in patients who have undergone peri-implant surgeries. The present study adds new and relevant data that will help clinicians in their choice of treatment. Patient-centered outcomes (OHRQoL and PSPSQ) are of the outmost importance and should be considered and discussed with patients before performing any treatment (9,16). The OHIP-14 questionnaire is a patient-centered tool designed to provide a comprehensive measurement of the dysfunction, discomfort and disability related to oral conditions. It provides information about functional limitations and treatment outcomes (17), thus giving an insight into the impact of surgery on the psychosocial well-being of the patient.

The present outcomes suggest that the effect on the patient’s quality of life after surgical treatment of peri-implantitis is similar to other oral surgical procedures (18,19). Reports on OHRQoL after periodontal surgery show that the greatest limitations occur during the first 2 days (20,21). Similarly, the patients in the present study reported higher pain levels during the first 72 hours, decreasing progressively from the fourth day.

Working participants reported significantly higher OHIP-14sp scores although sick leave was uncommon. The pain and disability could well have had an impact on the patient’s working life, which might explain the higher impact on their OHRQoL in comparison with participants who were not in employment. Likewise, the surgical approach also seems to be a relevant variable. Augmentation techniques (regenerative or combined approaches) had a higher impact on OHRQoL probably because of the more extensive flap manipulation and periosteal releasing incisions, which might cause additional pain and swelling.

Understanding the impact of peri-implant disease management on the OHRQoL is essential. Not only is information on patient-centered outcomes lacking, but at least 75% of patients seem to be unaware of the existence of peri-implant pathology (9,16), while many others have major misunderstandings or unrealistic expectations regarding dental implant treatment (22). Only 64% of patients with disease are concerned about peri-implantitis (16). This might be related to the fact that the impact of peri-implantitis on well-being seems to be relatively mild, as is its impact on the patient’s life (9,16). Therefore, it is important to explain the nature and consequences of peri-implantitis prior to implant therapy and the importance of regular maintenance to avoid peri-implant pathology.

Even though 80% of the patients were satisfied with the surgical treatment, more than one third of the participants (36.6%) would not repeat the treatment. This is probably related with the moderate pain experienced in the first 72 hours and with the impact on their quality of life.

The success rates of peri-implantitis treatment depend on multiple factors, but the progression of bone loss can be stopped or at least slowed down in the majority of the cases (23). Early treatment is justified by 2 observations: the prognosis for peri-implant surgery is better when performed at the initial stages of the disease (23,24), and bone loss progresses if left untreated (25). Although the disease itself seems to have a low impact on the patient’s well-being and life (22), its progression could lead to implant loss, which will affect the patient’s OHRQoL. When implant failure occurs, subsequent treatments might be difficult due to the presence of large bone defects caused by the removal of implants and by the disease itself. Consequently, the patient may require bone augmentation procedures, which have higher morbidity rates and entail longer healing times without the prosthesis. As a result, a major impact on the patient’s OHRQoL is highly likely, although no reports have addressed this issue or compared peri-implant surgery with bone augmentation procedures.

One limitation of this study is related to the limited experience of the clinicians who performed the peri-implant treatments, which might increase the impact on the OHRQoL, since more experienced surgeons are faster and are likely to achieve better outcomes. On the other hand, the fact that several clinicians were involved in the surgical procedures increases the external validity of these results. It is also important to stress that these results are limited to the first postoperative week and those variables like flap extension, implant position and the severity of the disease were not assessed. Further research should focus on the medium- and long-term impact of these treatments.

Surgical treatment of peri-implantitis has an impact on the patient’s OHRQoL, especially when the patient is in work or when regenerative or combined approaches are employed. Postoperative pain was mild to moderate in the first 72 hours, showing a steady decrease from the fourth postoperative day. Patient satisfaction was high despite one third of the patients’ not wishing to repeat the procedure.

## References

[1] Jemt T, Johansson J (2006). Implant treatment in the edentulous maxillae: a 15-year follow-up study on 76 consecutive patients provided with fixed prostheses. Clin Implant Dent Relat Res.

[2] Rakic M, Galindo-Moreno P, Monje A, Radovanovic S, Wang HL, Cochran D (2018). How frequent does peri-implantitis occur? A systematic review and meta-analysis. Clin Oral Investig.

[3] Lee CT, Huang YW, Zhu L, Weltman R (2017). Prevalences of peri-implantitis and peri-implant mucositis: systematic review and meta-analysis. J Dent.

[4] Schwarz F, Derks J, Monje A, Wang HL (2018). Peri-implantitis. J Periodontol.

[5] Romeo E, Ghisolfi M, Murgolo N, Chiapasco M, Lops D, Vogel G (2005). Therapy of peri-implantitis with resective surgery: A 3-year clinical trial on rough screw-shaped oral implants. Part I: Clinical outcome. Clin Oral Implants Res.

[6] Schwarz F, John G, Schmucker A, Sahm N, Becker J (2017). Combined surgical therapy of advanced peri-implantitis evaluating two methods of surface decontamination: a 7-year follow-up observation. J Clin Periodontol.

[7] Sischo L, Broder HL (2011). Oral health-related quality of life: what, why, how, and future implications. J Dent Res.

[8] Tan H, Peres KG, Peres MA (2016). Retention of teeth and oral health-related quality of life. J Dent Res.

[9] Romandini M, Lima C, Pedrinaci I, Araoz A, Costanza Soldini M, Sanz M (2021). Clinical signs, symptoms, perceptions, and impact on quality of life in patients suffering from peri-implant diseases: a university-representative cross-sectional study. Clin Oral Implants Res.

[10] von Elm E, Altman DG, Egger M, Pocock SJ, Gøtzsche PC, Vandenbroucke JP (2008). The Strengthening the Reporting of Observational Studies in Epidemiology (STROBE) statement: guidelines for reporting observational studies. J Clin Epidemiol.

[11] Berglundh T, Armitage G, Araujo MG, Avila-Ortiz G, Blanco J, Camargo PM (2018). Peri-implant diseases and conditions: Consensus report of workgroup 4 of the 2017 world workshop on the classification of periodontal and peri-implant diseases and conditions. J Periodontol.

[12] de Tapia B, Mozas C, Valles C, Nart J, Sanz M, Herrera D (2019). Adjunctive effect of modifying the implant-supported prosthesis in the treatment of peri-implant mucositis. J Clin Periodontol.

[13] Schwarz F, Herten M, Sager M, Bieling K, Sculean A, Becker J (2007). Comparison of naturally occurring and ligature-induced peri-implantitis bone defects in humans and dogs. Clin Oral Implants Res.

[14] Montero-Martín J, Bravo-Pérez M, Albaladejo-Martínez A, Hernández-Martín LA, Rosel-Gallardo EM (2009). Validation the Oral Health Impact Profile (OHIP-14sp) for adults in Spain. Med Oral Patol Oral Cir Bucal.

[15] Graetz C, Schwalbach M, Seidel M, Geiken A, Schwendicke F (2020). Oral health-related quality of life impacts are low 27 years after periodontal therapy. J Clin Periodontol.

[16] Insua A, Monje A, Wang HL, Inglehart M (2017). Patient-centered perspectives and understanding of peri-implantitis. J Periodontol.

[17] Locker D, Matear D, Stephens M, Lawrence H, Payne B (2001). Comparison of the GOHAI and OHIP-14 as measures of the oral health-related quality of life of the elderly. Community Dent Oral Epidemiol.

[18] Stefanini M, Tavelli L, Barootchi S, Sangiorgi M, Zucchelli G (2021). Patient-reported outcome measures following soft-tissue grafting at implant sites: A systematic review. Clin Oral Implants Res.

[19] Starch-Jensen T, Ahmad M, Bruun NH, Becktor JP (2021). Patient's perception of recovery after maxillary sinus floor augmentation with autogenous bone graft compared with composite grafts: a single-blinded randomized controlled trial. Int J Implant Dent.

[20] Hagenaars S, Louwerse PHG, Timmerman MF, Van der Velden U, Van der Weijden GA (2004). Soft-tissue wound healing following periodontal surgery and Emdogain application. J Clin Periodontol.

[21] Ozcelik O, Haytac MC, Seydaoglu G (2007). Immediate post-operative effects of different periodontal treatment modalities on oral health-related quality of life: a randomized clinical trial. J Clin Periodontol.

[22] Abrahamsson KH, Wennström JL, Berglundh T, Abrahamsson I (2017). Altered expectations on dental implant therapy; views of patients referred for treatment of peri-implantitis. Clin Oral Implants Res.

[23] Serino G, Turri A (2011). Outcome of surgical treatment of peri-implantitis: Results from a 2-year prospective clinical study in humans. Clin Oral Implants Res.

[24] Froum SJ, Rosen PS, Wang WC, Froum SH, Vinayak S (2018). Retrospective evaluation of factors related to the outcomes of regenerative therapy for implants affected by peri-implantitis. Int J Periodontics Restorative Dent.

[25] Derks J, Schaller D, Håkansson J, Wennström JL, Tomasi C, Berglundh T (2016). Peri-implantitis - onset and pattern of progression. J Clin Periodontol.

